# Over-Expression of *ERF38* Gene Enhances Salt and Osmotic Tolerance in Transgenic Poplar

**DOI:** 10.3389/fpls.2019.01375

**Published:** 2019-11-04

**Authors:** Zihan Cheng, Xuemei Zhang, Kai Zhao, Wenjing Yao, Renhua Li, Boru Zhou, Tingbo Jiang

**Affiliations:** ^1^State Key Laboratory of Tree Genetics and Breeding, Northeast Forestry University, Harbin, China; ^2^Bamboo Research Institute, Nanjing Forestry University, Nanjing, China

**Keywords:** poplar, *ERF38* gene, salt and osmotic tolerance, gene expression, genetic transformation

## Abstract

Ethylene response factor (ERF) gene family plays an important role in abiotic stress responses. In this study, we isolated a salt-inducible ERF gene, *ERF38* (Potri.006G138900.1), from the 84K poplar (*Populus alba × Populus glandulosa*) and investigated its functions in salt and osmotic tolerance. We identified that ERF38 protein was targeted to nucleus and had no self-activation. Results from yeast-one-hybrid indicated that the ERF38 protein can specifically bind to the dehydration responsive element (DRE). We then successfully transferred the *ERF38* gene into the 84K poplar. Under respective salt and polyethylene glycol (PEG)-6000 stresses, four of the physiological traits, including peroxidase (POD) and superoxide dismutase (SOD) activities, soluble protein content, and proline content, increased significantly in the transgenic plants, compared to the wild type. Regarding the other two parameters, hydrogen peroxide (H_2_O_2_) and malondialdehyde (MDA) content, their increments in the transgenic lines under the stresses, which were compared to the water control, were significantly low than that of the wild type. In addition, reactive oxygen species (ROS) are scavenged in the transgenic lines under the stresses, but not in the wild type (WT). Interestingly, when challenged with the stresses, expression levels of a few genes associated with POD and SOD metabolism were significantly increased in the transgenic poplars. In all, evidence from morphological, physiological, and biochemical analyses indicated that over-expression of *ERF38* gene can improve salt and osmotic tolerance in the transgenic poplar.

## Introduction

Abiotic stresses, such as high salt, drought, and low temperature, seriously affect plant growth and development. Upon environmental stresses, plants sense the changes and transmit corresponding signal pathways, which result in activating or repressing expression of related genes. Expression of these genes can lead to a series of morphological, physiological, and biochemical reactions in response to changes of the stresses. For instance, plants can regulate the closure of the stomata to control the loss of water, thus alleviating salt or drought damage (He et al., 2018; Zhang et al., 2019). Furthermore, antioxidant enzymes in plants, including superoxide dismutase (SOD), peroxidase (POD), glutathione reductase (GR), will increase correspondingly, in order to reduce the contents of reactive oxygen species (ROS) that are induced by abiotic stresses (Scandalios, 2005; Yu et al., 2017; Zhu et al., 2019).

Transcription factors (TFs) impact plant stress responses significantly. TFs can regulate expression of their target genes by binding to specific cis-elements of the genes, in order to respond to the abiotic stresses (Singh et al., 2002; Chen and Zhu, 2004). Many studies indicated that through various mechanisms over-expression of several TF genes can enhance tolerance to abiotic stress. For example, over-expression of tomato *SlNAC35* gene in transgenic tobacco increase root length, which leads to enhanced salt and drought tolerance (Wang et al., 2016). Over-expression of *OsWR1* gene can increase wax content and enhance drought resistance of transgenic rice by reducing water loss (Wang et al., 2012). In addition, Zhang and colleagues found that over-expression of *LeERF2/TERF2* gene in tobacco and tomato can enhance the expression of ethylene synthesis gene, thereby activating the expression of some low-temperature related genes and enhancing the cold resistance of plants (Zhang and Huang, 2010).

As one of the largest plant-specific TFs, the ethylene response factor (ERF) family plays an important role in plant growth and development (Li et al., 2018a), lignification (Ma et al., 2017), seed germination (Li et al., 2017), and plant defense (Onate-Sanchez et al., 2007). Moreover, many proteins from the ERF family are able to recognize specific deoxyribonucleic acid (DNA) motif sequences, such as the dehydration responsive element (DRE) motifs (GCCGAC) and the GCC-box (AGCCGCC). For instance, the protein encoded by *gmERF3* gene can bind to the GCC-box and the DRE/C-repeat motifs, in order to regulate target gene expression (Zhang et al., 2009). In transgenic *Arabidopsis*, the protein encoded by *MintMaRAP2-4* gene can regulate water logging by binding to various regulatory elements, such as the DRE motif, the jasmonic acid response element (GATGAATT) motif, and the GCC-box (Phukan et al., 2018). Furthermore, ERF genes play an indispensable role in plant responses to abiotic stresses. For instance, over-expression of tomato *SlERF84* gene in *Arabidopsis* can increase salt tolerance (Li et al., 2018b). In wheat, the *TaERF3* gene can increase salt and drought tolerance of transgenic wheat (Rong et al., 2014). Our previous studies indicated that transgenic poplars over-expressing *ERF76* gene are superior to wild type (WT) in morphological and physiological traits under salt stress (Yao et al., 2016).

The poplar variety 84K is a hybrid strain from the cross of *Populus alba* and *Populus glandulosa*. In our previous studies, we found that poplar *ERF38* gene had a high expression under salt stress (Yao et al., 2018). Thus we speculated that the poplar *ERF38* gene play an important role in the response to abiotic stresses. We then successfully cloned the gene and genetically transformed it into the 84K poplar. Compared to the WT, the transgenic plants showed better physiological and morphological traits, and displayed superior tolerance to salt and osmotic stresses.

## Materials and Methods

### Poplar Materials and Growth Conditions

One-month-old poplar WT seedlings from 84K variety were grown with hydroponic culture for 30 days, without nutrients. Poplar seedlings with new leaves and roots were treated with 150 mM sodium chloride (NaCl) and 20% PEG-6000, respectively. Samples from young leaves, stems, and roots were collected at each time point of 0, 3, 6, 12, 24, and 48 h, with three biological replicates. Then the samples were frozen in liquid nitrogen and stored at -80°C in refrigerator.

Sterile poplar seedlings were grown on 1/2 Murashige and Skoog medium (MS), followed by rooting medium containing 0.01 mg/ml 1-naphthaleneacetic acid (NAA) and 0.1 mg/ml indole-3-butyric acid (IBA). Poplar leaves from 1-month-old seedlings were cut into 1 cm x 1 cm leaf disks and place on MS differentiation medium containing 0.1 mg/ml NAA and 0.04 mg/ml thidiazuron (TDZ) for differentiation. All materials were grown in a greenhouse at 25°C with 16/8-h light/dark cycles.

### Phylogenetic Analysis of Ethylene Response Factor Genes

Protein sequences of the ERF genes from nine different species were obtained from the National Center for Biotechnology Information database (http://www.ncbi.nlm.nih.gov/). Multiple sequence alignment was performed by use of clustalW (Thompson et al., 1994). Phylogenetic tree analysis was conducted by MEGA 6 (Tamura et al., 2013), using neighbor-joining method.

### Cloning of *ERF38* Gene

The coding sequence of *ERF38* gene (Potri.006G138900.1) from *Populus trichocarpa* was employed to design primers. The complementary deoxyribonucleic acid (cDNA) of *ERF38* gene was inserted into the pBI121 binary vector with *XbaI* and *SacI* restriction sites. The recombination vector was then transferred into *Agrobacterium* GV3101 by the freezing and thawing method (Holsters et al., 1978), followed by infection and transformation with *Agrobacterium tumefaciens*.

### Transcriptional Activation and Yeast One-Hybrid Assays

Transcriptional activation of the ERF38 protein was tested. Primers with *EcoRI* and *BamHI* restriction sites were designed to insert the full-length coding sequence of *ERF38* gene into pGBKT7 to form pGBKT7-ERF38 fusion vector containing GAL4-DNA domain. The pGBKT7-ERF38 fusion vector, pGBKT7 vector (negative control), and pGBKT7-53/pGADT7-T (positive control) were transferred into yeast two-hybrid (Y_2_H) yeast cells, respectively. Then they were cultured on the SD/-Trp or SD/-Trp/-His/X-a-Gal medium at 30°C for 3–5 days.

The cDNA fragment of *ERF38* was inserted into pGADT7 vector to form pGADT7-ERF38. The three tandem repeats of the DRE (GCCGAC) motif, and two mutant elements, M1 (GTCGGC) and M2 (GTTTAC), were combined to pAbAi to form bait reporter vectors, respectively. Respective reporters and p53-AbAi (negative control) vectors were transferred to yeast-one-hybrid yeast strain, followed by culture on SD/-Ura medium for 3–5 days. Positive clones were selected by small-scale transformation in the yeast transformation system. The transformations were diluted to 10, 100, and 1,000 times, respectively; and then spread on SD/-Leu and SD/-Leu/AbA plates.

### Subcellular Localization

The coding region of the *ERF38* cDNA without stop codon was inserted into pBI121 vector containing green fluorescent protein (GFP), and driven by the 35S promoter, i order to form a 35S::ERF38::GFP fusion vector. The empty vector was used as a control (35S::GFP). Respective fusion vectors and 35S::GFP were transiently transformed into epithelial cells of the onions by particle bombardment. And the signal of GFP was observed under a laser confocal scanning microscope (LSM 700, Zeiss, Germany). Excitation wavelength used in 488 nm for GFP, and the wavelength range of captured light at 515–555 nm.

### Generation of Transgenic Poplar

The transgenic poplar was obtained by leaf disk method (Nehra et al., 1990; Nietsch et al., 2017). Poplar leaves from 1-month-old seedlings were cut to 1 cm x 1 cm leaf disks and put on MS differentiation medium containing 50 mg/L kanamycin and 200 mg/L cephalosporin. The seedlings were then grown on rooting medium containing 50 mg/L kanamycin. The specific primers were designed to detect the positive transgenic poplars by both polymerase chain reaction (PCR) and reverse transcriptase PCR (RT-PCR).

### Morphological and Physiological Measurements

The transgenic poplars were cultured for one month in rooting medium containing 0, 35, 50, 100 mM NaCl, respectively. Plant height, root length, and fresh weight of the plants at 1-month-old were measured with three biological replicates.

The seedlings at 1-month-old were transplanted into the soil in a greenhouse for another month, then they were treated with 150 mM NaCl (5 days) and 20% PEG-6000 (5 days), respectively. The morphological traits of both transgenic poplar and WT seedlings under normal and stress conditions were observed. Physiological parameters were measured, such as contents of proline, SOD, POD, H_2_O_2_, malondialdehyde (MDA), and soluble protein of transgenic poplar and WT (Yao et al., 2016; Wang et al., 2017). Each sample had three biological replicates.

### Histochemical Staining and Gene Expression Analysis

Both 3,3′-diaminobenzidine (DAB) and nitrotetrazolium blue chloride (NBT) staining were used to detect the activities of hydrogen peroxide and superoxide in plants (Kumar et al., 2013). Quantitative real-time PCR (RT-qPCR) was used to detect the relative expression levels of POD and SOD-related genes in transgenic plants under salt and PEG-6000 stresses (Zhang et al., 2019).

### Reverse Transcriptase Quantitative Polymerase Chain Reaction

Total ribonucleic acid (RNA) from 84K seedlings was extracted by the RNA Isolation Kit (TAKALA, Dalian), and was reversely transcribed into cDNA using the reverse transcription kit (TAKALA, Dalian). The cDNA concentration of each sample was uniformly adjusted to the 100 ng/µl. RT-qPCR was performed using the Power SYBR Green Master mix method of ABI7500 Real Time System. The procedures include 95.0°C for 30 s, 95.0°C for 5 s, 60.0°C for 34 s, 95.0°C for 15 s, 60.0°C for 1 min, and 95.0°C for 15 s. The data was processed using 2^-∆∆Ct^ method (Dong et al., 2015). Each sample had three biological replicates. All primer sequences used in this study were shown in **Supplementary Table S1 and S2**.

## Results

### Alignment and Phylogenetic Tree Analysis of Ethylene Response Factor Proteins

**Figure 1:**
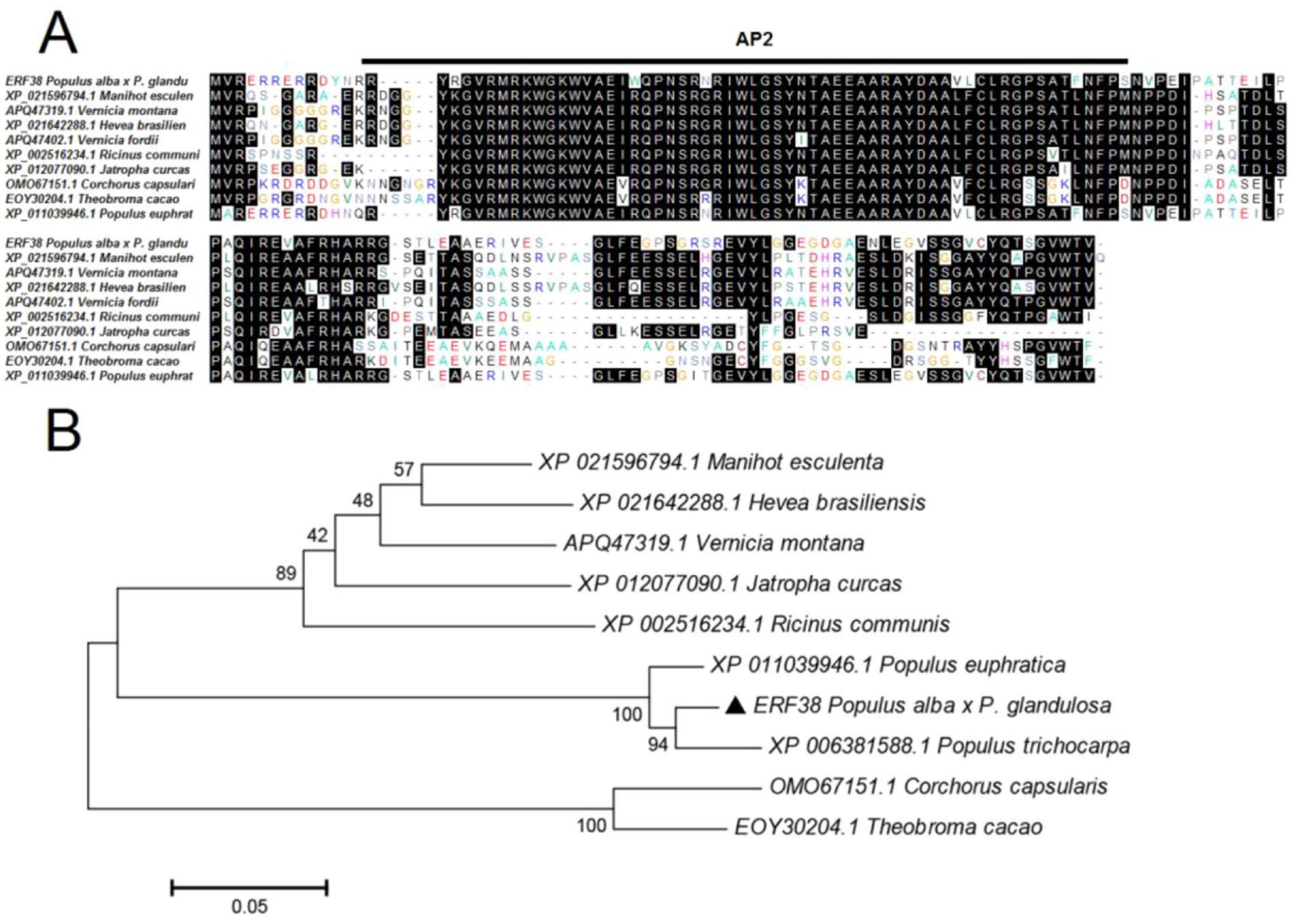
Analysis of ERF38 and homologous protein sequences. **(A)** Alignment of amino acid sequences of ERF38 and ethylene response factors (ERFs) from multiple species. **(B)** Phylogenetic analysis of ERF38 and the ERF family proteins from the species.

Comparisons of protein sequences from different species can help us to understand their phylogenetic relationships. The 465-bp cDNA fragment of *ERF38* gene was cloned from 84K poplar. Sequence similarity of ERF38 with the proteins from *Populus trechocarpa*, *Populus euphratica*, *Vernicia montana*, *Manihot esculenta*, *Ricinus communis* was 97.14, 94.16, 63.35, 65.10, and 61.33%, respectively. Evidence from multiple sequences alignment indicated that all proteins shared a conserved domain named as AP2 (**Figure 1A**). In addition, phylogenetic tree analysis demonstrated that the ERF38 protein from 84K poplar was closely related to the counterparts from *P. trechocarpa* and *P. euphratica*, but had a distant genetic relationship with the proteins from *Corchorus capsularis*, *Theobroma cacao*, and *M. esculenta* (**Figure 1B**).

### Tempo-Spatial Expression Pattern of *ERF38* Gene

**Figure 2:**
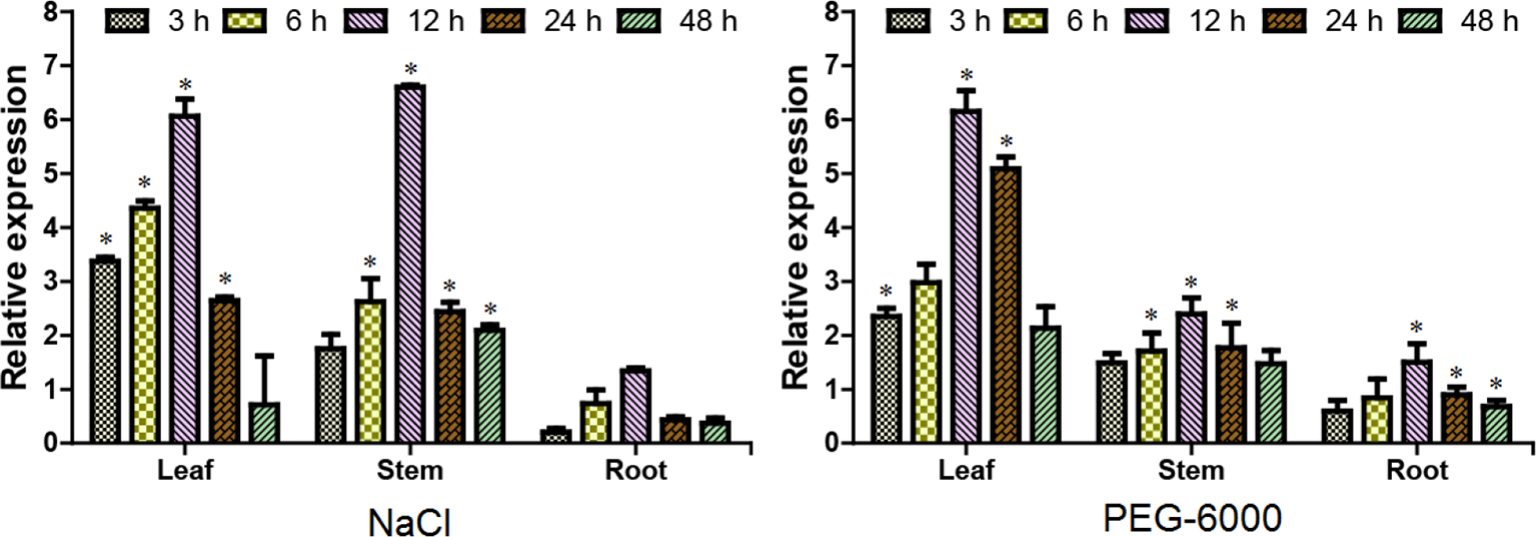
Tempo-spatial expression patterns of the *ERF38* gene under stresses. After 150 mM NaCl and 20% polyethylene glycol-6000 treatments at each time point of 0, 3, 6, 12, 24, and 48 h, the roots, stems, and leaves were collected and used for reverse transcriptase quantitative polymerase chain reaction. The data was processed using the 2^-ΔΔCt^ method. The error bars represent the standard deviation. Asterisks indicate significant differences between transgenic lines and wild type lines (t test, *P < 0.05).

Evidence from gene expression analysis indicated that *ERF38* gene displayed a tempo-spatial pattern that is similar to NaCl and PEG stresses (**Figure 2**). It is clear that *ERF38* gene is mainly expressed in leaves and stems, but relative lowly expressed in roots. And the relative expression level of *ERF38* gene reached a peak at 12 h, followed by a decrease at 24 h (**Figure 2**).

### ERF38 Protein Was Localized to the Nucleus

**Figure 3:**
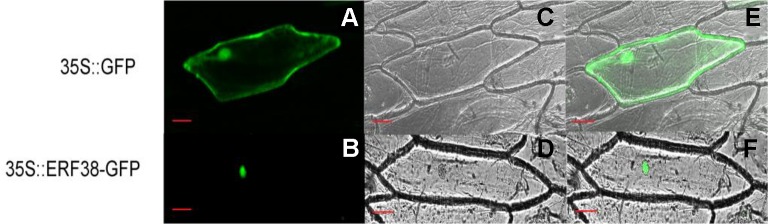
Subcellular localization of ERF38 protein. **(A)** and **(D)** are dark field images, **(B)** and **(E)** are bright field images, **(C)** and **(F)** are images of dark field and bright field. Scale bar = 50 μm.

As shown in **Figure 3**, the fluorescence signal of 35S::ERF38::GFP only appears in the nucleus, while the 35S::GFP appeared in the cytoplasm, nucleus and cell membrane. This indicates that the ERF38 protein is localized to the nucleus of plant cell.

### ERF38 Protein Had No Self-Activation and Specifically Bind to the Dehydration Responsive Element Motif

**Figure 4:**
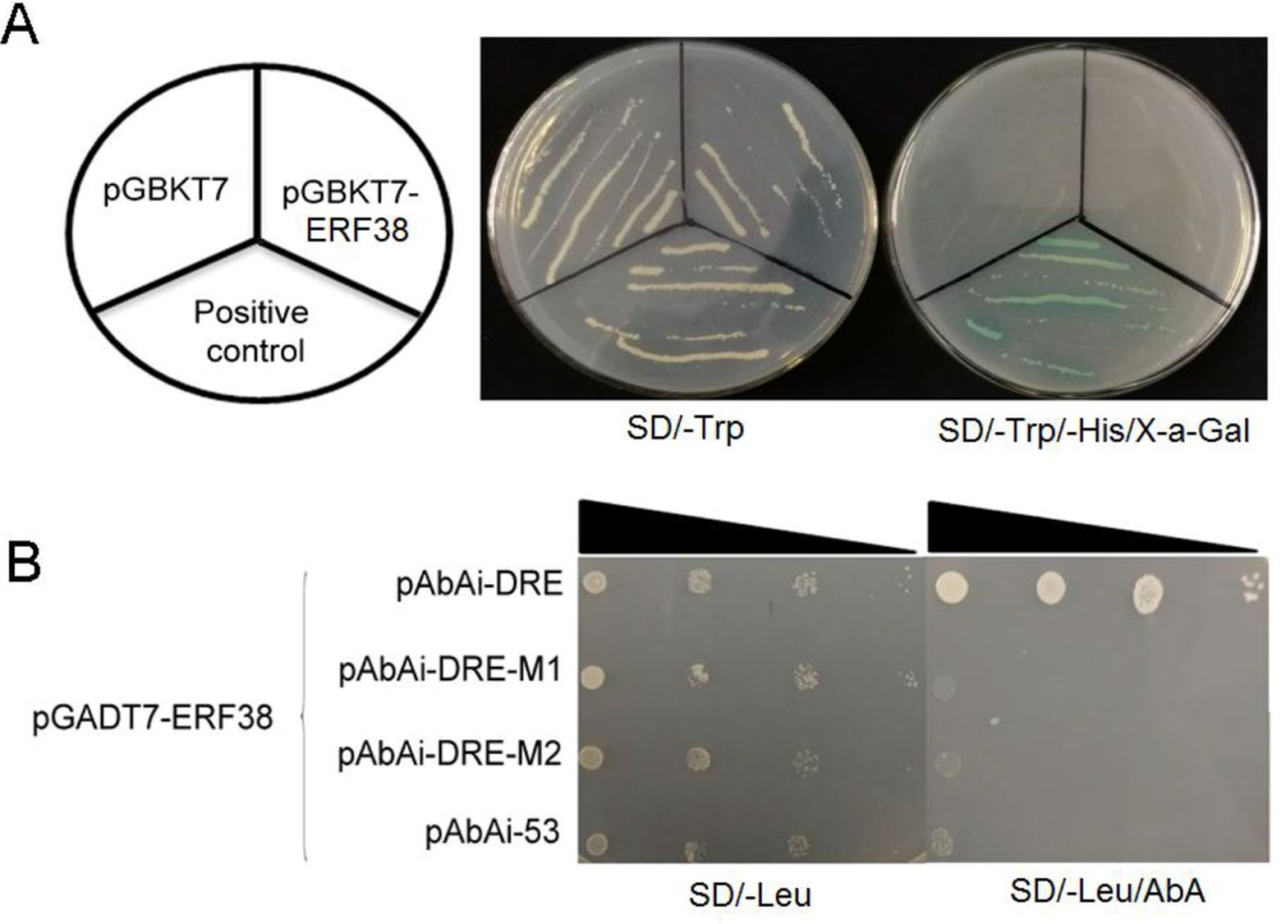
Transcription activation activity assay and protein-deoxyribonucleic acid interaction analysis. **(A)** All three strains are able to grow normally on SD/-Trp medium, but only positive control can grow and turn blue on the SD/-Trp/-His/X-a-Gal medium. **(B)** Yeast one-hybrid assay was used to verify ERF38 protein specifically binding to the dehydration responsive element (DRE) motif, but not to the two DRE mutants.

The coding sequence of *ERF38* was inserted into pGBKT7 *in vivo*, and transformed into the Y_2_H with a negative control and a positive control. As shown in **Figure 4A**, pGBKT7-ERF38 is able to grow normally on the SD/-Trp medium, as with the positive and negative controls. The positive control grew normally and turned blue on the SD/-Trp/-His/X-a-Gal medium, however, both pGBKT7-ERF38 and the negative control did not grow, indicating that the ERF38 protein has no self-activation.

Yeast one-hybrid was conducted to verify whether the ERF38 protein binds to the DRE element. Results indicated that the yeast cells were able to grow normally on the SD/-Leu medium, but not on the SD/-Leu medium containing 100 mg/ml AbA. Only the yeast strain, harboring ERF38 protein binding with the DRE motif, can grow on the SD/-Leu/AbA. Negative control and other mutant motifs cannot grow normally with AbA (**Figure 4B**). Therefore, it appears that ERF38 can specifically bind to the DRE motif.

### Molecular Identification of Transgenic Plants

**Figure 5:**
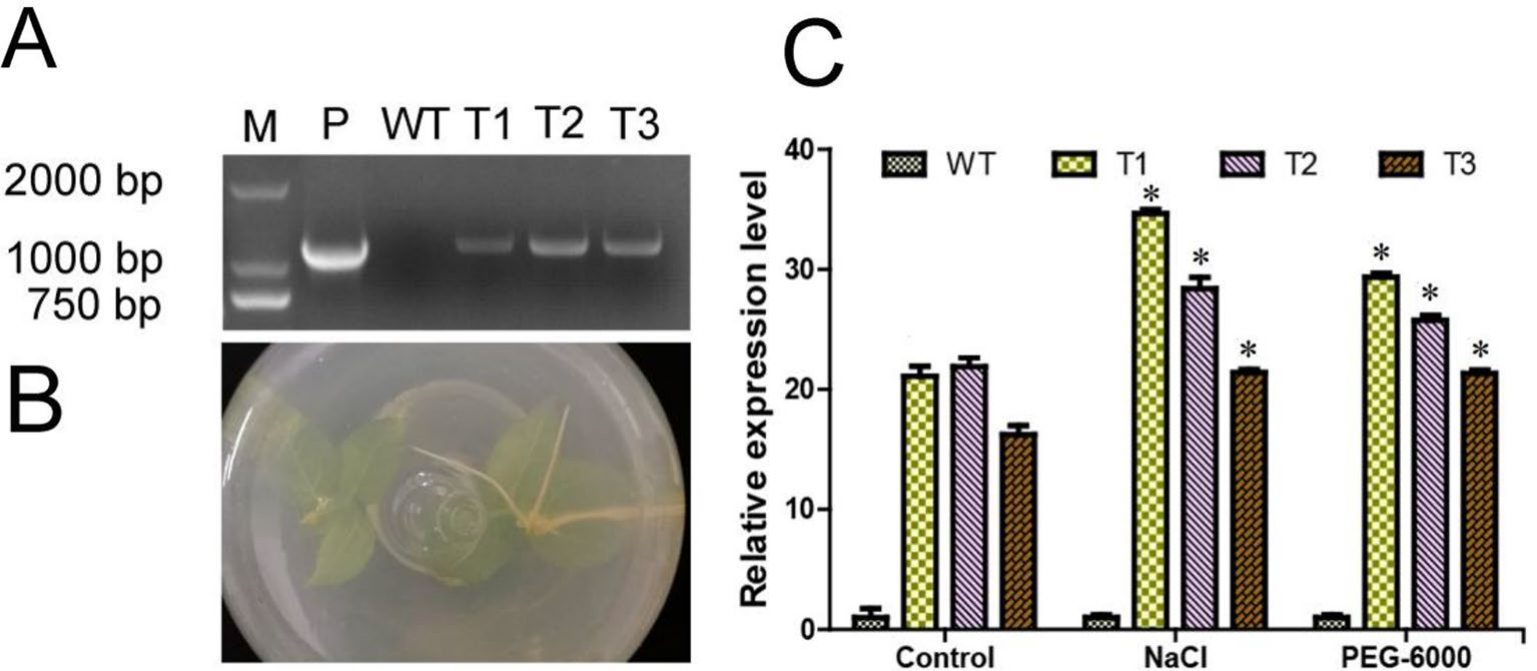
Identification and expression analysis of transgenic poplar. T1–T3: different transgenic poplar lines; WT, the wild type poplar. **(A)** Identification of poplar transgenic lines; M, 2000 DNA maker; P, positive control with recombined vector as the template. **(B)** Transgenic lines can root in the rooting medium containing 50 mg/ml kanamycin. WT seeding is on the left, transgenic seedling is on the right. **(C)** Relative expression level of *ERF38* gene in the WT and transgenic lines by reverse transcriptase quantitative polymerase chain reaction. The error bars represent the standard deviation. Asterisks indicate significant differences between transgenic lines and wild type lines (t test, *P < 0.05).

As shown in **Figure 5A**, the exogenous *ERF38* fragment was only identified in the transgenic poplar lines, but not in the WT seedlings. The transgenic seedlings can grow root normally in the rooting medium containing 50 mg/ml kanamycin and 200 mg/ml cephalosporin, however, the WT seedlings cannot grow root in the same medium (**Figure 5B**). The relative expression level of *ERF38* in transgenic poplar lines was significantly higher than that in the WT under both normal and stress conditions (**Figure 5C**).

### Morphological Changes in Response to Abiotic Stresses

**Figure 6:**
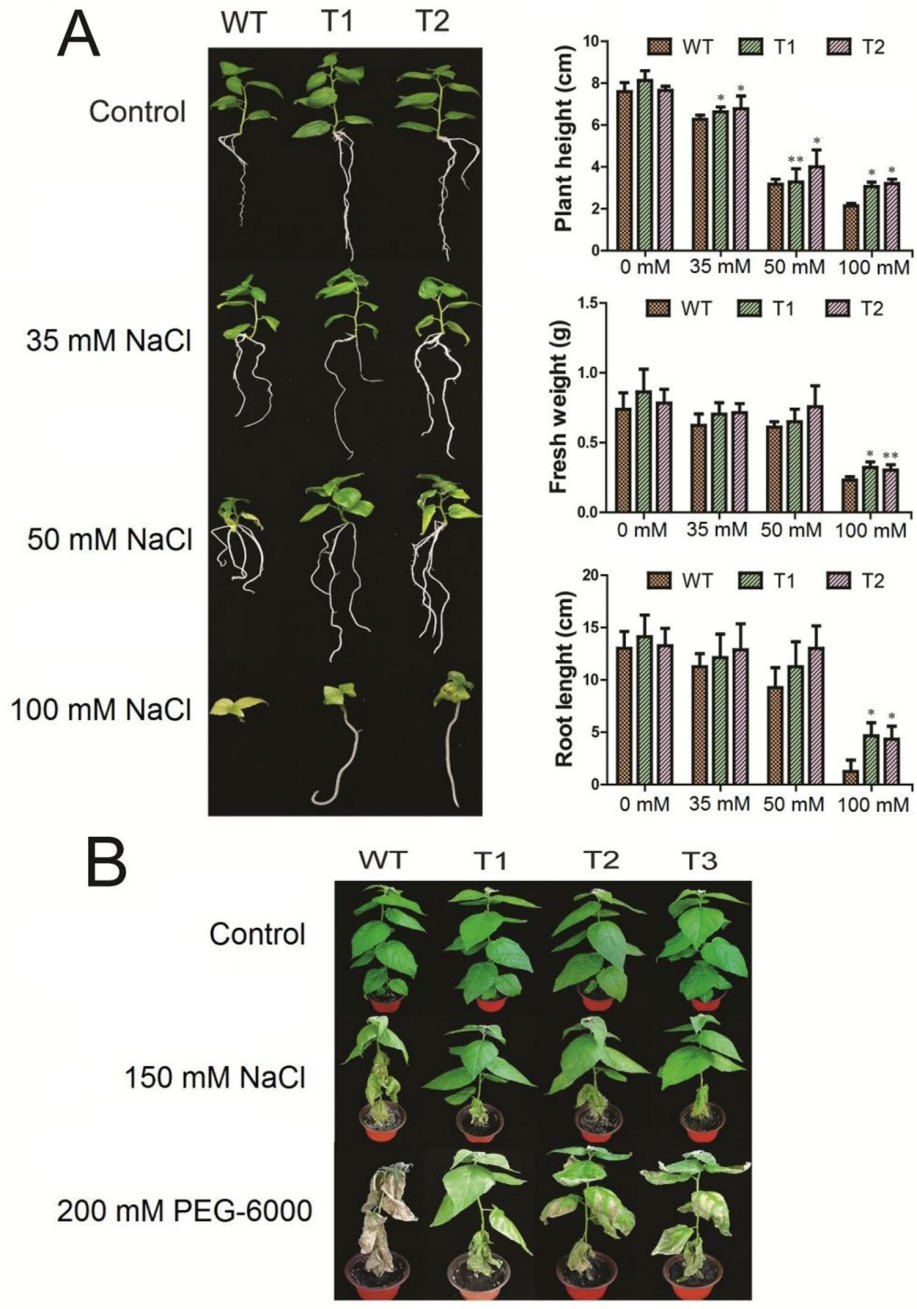
Morphological characteristics of transgenic poplars under two stresses. T1–T3: different transgenic poplar lines; WT, the wild type 84K poplar. **(A)** Phenotype of poplars at 1-month-old in the rooting medium containing 0, 35, 50, and 100 mM NaCl, respectively. Plant height, root length, and fresh weight of both the transgenic and WT seedlings under the salt stresses. **(B)** The WT and transgenic plants at one-month-old were treated with respective 150 mM salt and PEG-6000 for 5 days. The error bars represent standard deviation. Asterisks indicate significant differences between transgenic lines and wild type lines (t test, *P < 0.05, **P < 0.01).

Seedlings from two transgenic lines and the WT were treated with 0, 35, 50, and 100 mM NaCl, respectively. Our data showed that under normal conditions, the plant height, root length, and fresh weight of transgenic poplar were 1.04, 1.12, and 1.05 times than that of WT, respectively. Under 35 mM NaCl conditions, the plant height, root length, and fresh weight of transgenic poplars were 1.07, 1.14, 1.11 times than those of WT, respectively. Under 50 mM NaCl conditions, the values were 1.15, 1.13, 1.15 times, respectively. And under 100 mM NaCl conditions, the ratio changed to 1.47, 3.60, 1.36 times compared to WT, respectively (**Figure 6A**).

To compare the transgenic lines and the WT under both abiotic stresses, we treated the 1-month-old plants with respective 150 mM salt and 20% PEG-6000 for 1 week. As shown in **Figure 6B**, the transgenic poplar and WT grew normally and there was no morphological difference under the control condition. Under the salt stress, however, the WT seedlings became wilting with dropping leaves, while the transgenic lines showed no obvious symptom (**Figure 6B**). In contrast, under the PEG-6000 stress, the WT plant died, while the transgenic lines still survive with observable symptoms (**Figure 6B**).

### Physiological Changes and Gene Expression in Response to Both Salt and Osmotic Stresses

**Figure 7:**
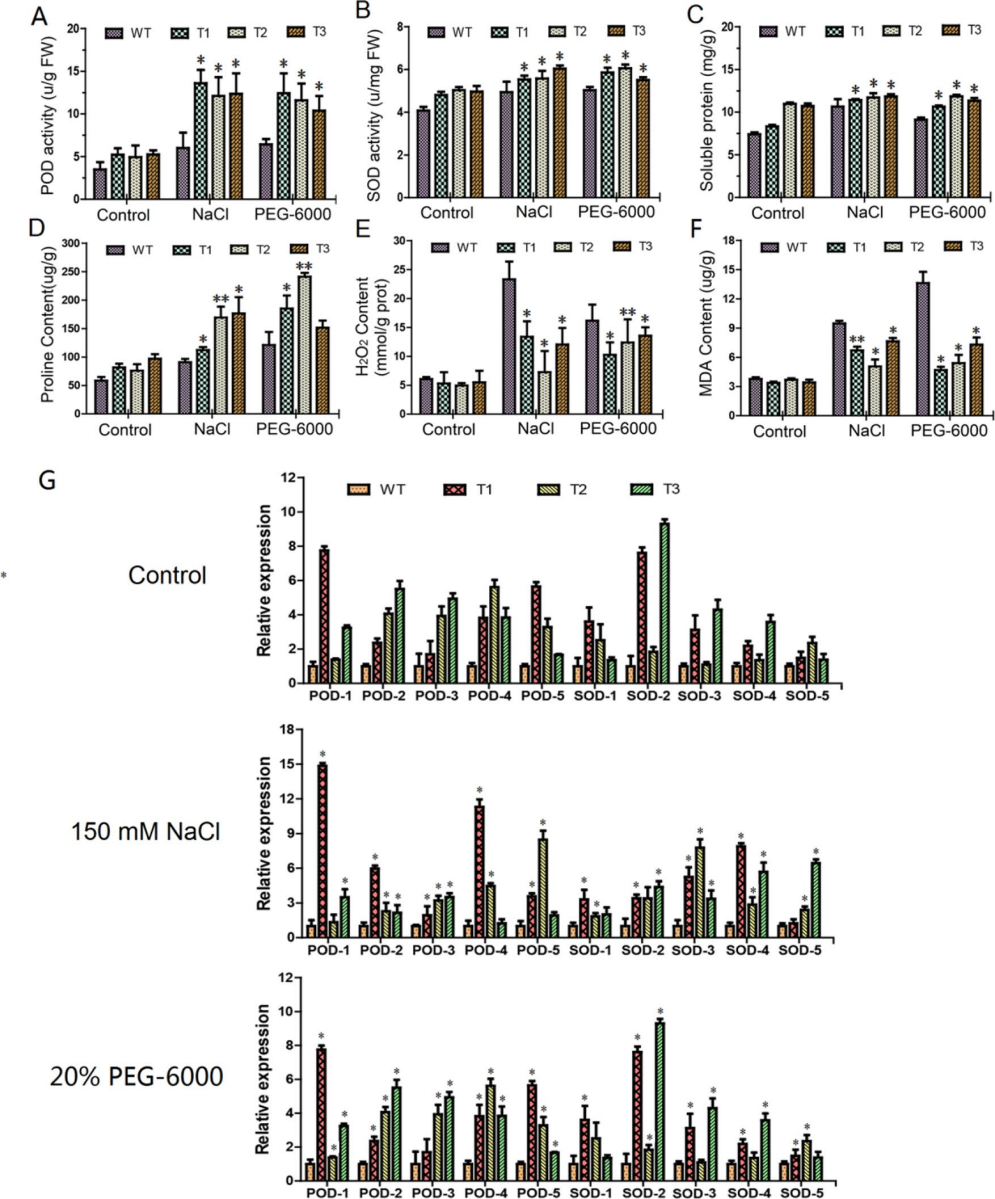
Analysis of physiological and gene expression under both salt and polyethylene glycol stresses. T1–T3: different transgenic poplar lines; WT, wild type poplar. **(A**–**F)** Comparison of peroxidase (POD), superoxide dismutase (SOD), proline, soluble protein, H_2_O_2_, and malondialdehyde contents between transgenic lines and the WT; the control is water. **(G)** Expression of POD and SOD-related genes in transgenic plants under salt and PEG-6000 stresses. The error bars represent the standard deviation. Asterisks indicate significant differences between transgenic lines and wild type lines (t test, *P < 0.05, **P < 0.01)

We measured six physiological parameters of both the transgenic lines and the WT, under the two abiotic stresses and a water control (**Figures 7A–F**). In general, the two stresses displayed a similar physiological profiling pattern, with significant increases in the measurements, compared to the water control. Under the control condition, physiological traits, including POD and SOD activities, soluble protein content, and proline content, were significantly higher in the transgenic poplar than that in the WT (**Figures 7A–D**). But transgenic lines and the WT displayed no significant difference in MDA and H_2_O_2_ contents (**Figures 7E, F**). Under each of the stresses, four of the physiological traits, including POD and SOD activity, soluble protein content, and proline content, increased significantly in the transgenic plants, compared to the WT (**Figures 7A–D**). Regarding the other two physiological traits (H_2_O_2_ and MDA content), the increments in the transgenic lines under the stresses, which is compared to the water control, were significantly low than that of the WT, suggesting an improved ROS environment in the transgenic lines, compared to the WT (**Figures 7E, F**).

Furthermore, relative expression levels of the genes relevant to POD and SOD metabolism in the transgenic lines and the WT were measured by RT-qPCR. Under the control condition, the expression levels of such five genes were significantly higher in the transgenic lines than that in the WT (**Figure 7G**). Similarly, under salt and PEG-6000 stress conditions, their expression levels were still higher in the transgenic lines than that in the WT (**Figure 7G**). These lines of evidence indicated that *ERF38* gene may regulate the expression of the POD and SOD-related genes.

Similarly, we measured relative expression levels of four abscisic acid (ABA)-responsive genes (*RD29B*, *ZmRD22B*, *PtPYRL1*, and *ZmPTF1*) in the transgenic lines and the WT under normal growth and PEG-6000 stress conditions by RT-qPCR. These genes all can increase the plant tolerance by participating in the ABA signaling pathway (Yamaguchi-Shinozaki and Shinozaki, 1994; Yu et al., 2016; Phillips and Ludidi, 2017; Li et al., 2019b). Our results displayed that under the control condition, the expression levels of genes had no significant difference (**Supplementary Figure S1**). However, under the PEG-6000 condition, the expression of genes was significantly lower in the WT than in the transgenic poplars (**Supplementary Figure S1**).

### Histochemical Staining

**Figure 8:**
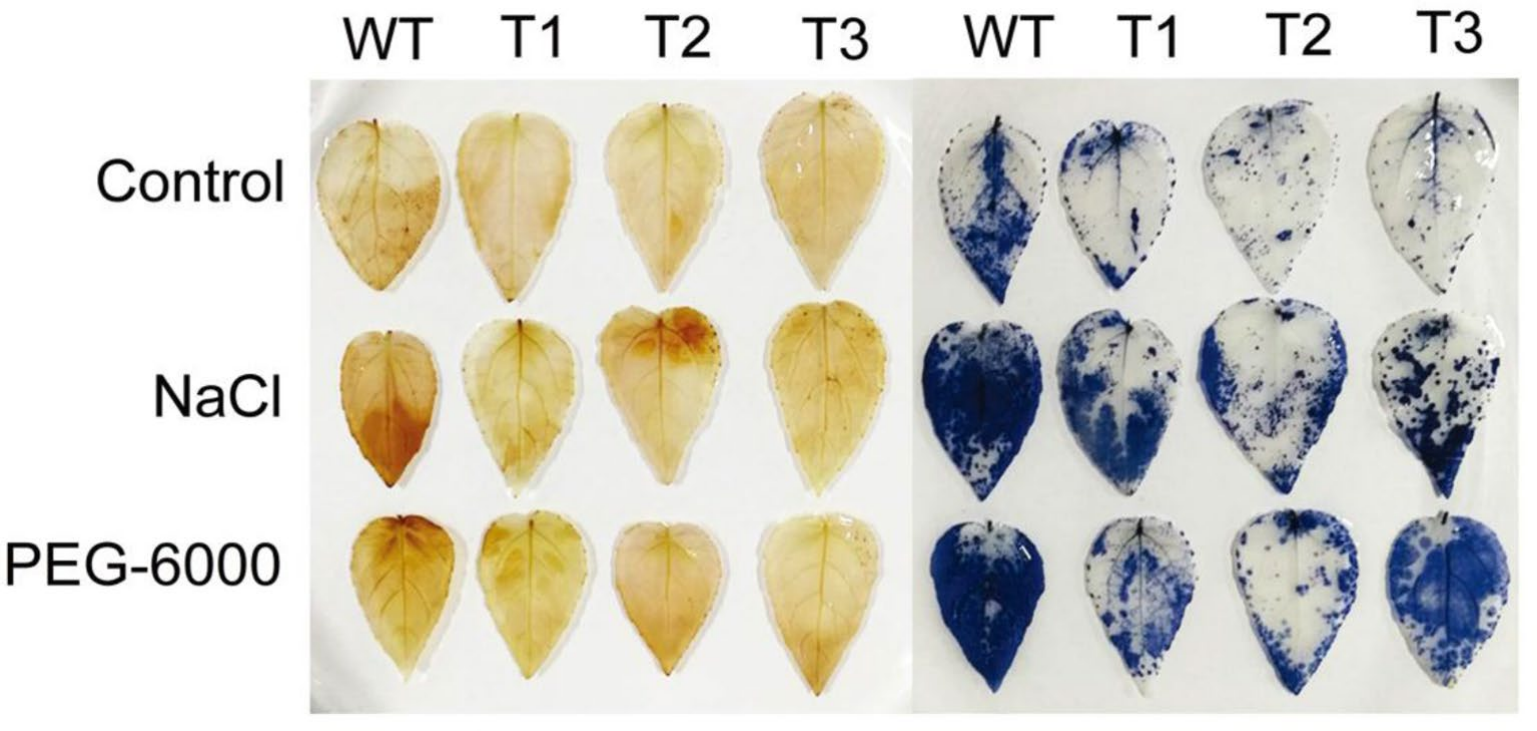
Analysis of reactive oxygen species scavenging mediated by *ERF38*. Nitrotetrazolium blue chloride and 3,3′-diaminobenzidine staining were used to detect H_2_O_2_ and O^2-^ contents, respectively.

Both NBT and DAB staining were used to detect the contents of hydrogen peroxide and superoxide, respectively. As shown in **Figure 8**, there was no significant difference in NBT staining between the transgenic seedlings and the WT under the control condition. After salt and PEG-6000 treatments, however, staining area of the transgenic seedlings was significantly smaller than that of the WT seedlings. Similar trend was observed for the DAB staining (**Figure 8**). These results indicate that accumulation of ROS in the transgenic lines was lower than that in WT.

## Discussion

ERF transcription factors play an important role in regulation of responses to abiotic stresses in plants. ERFs have been isolated from various plant, such as *Arabidopsis thaliana* (Bolt et al., 2017), *tomato* (Pan et al., 2012), *rice* (Jisha et al., 2015), *wheat* (Mondini et al., 2015). In this study, we identified a salt-induced and nucleus-targeted gene from the ERF gene family, *ERF38*. The gene encodes 155 amino acids, with the 14th amino acid in the conserved domain of valine acid, which indicates that *ERF38* gene belongs to DREB subfamily. Evidences from Y_2_H indicated that ERF38 protein is not self-activating, and it also can specifically bind to the DRE element.

Many ERF transcription factors can alter the morphological characteristics of plants. For example, over-expression of *ERF76* gene in poplar changed leaf type, and increased root length and plant height of transgenic poplar (Yao et al., 2016). Over-expressing of chrysanthemum *CmERF053* gene increased lateral buds and adventitious roots of transgenic chrysanthemums (Nie et al., 2018). In our study, we verified that the expression of *ERF38* gene was significantly inducible after high salt and osmotic stress treatments. And the transgenic poplars over-expressing *ERF38* gene displayed morphological advantages, compared to the WT poplars.

High salt and drought are main abiotic stresses threatened plant growth and development. There will be large amounts of reactive oxygen free radicals (including O_2_^-^, H_2_O_2_, hydroxide anions, etc.) accumulated in plants under the two stress conditions. Excessive ROS have a strong toxic effect on macromolecular substances, such as plant cell membrane systems, proteins and nucleic acids (Sarvajeet Singh and Narendra, 2010; Zhang et al., 2011). MDA content is the embodiment of the degree of membrane peroxidation in plant cells, and the higher its content, the more serious damage to the plant cell membrane (Tamirisa et al., 2014; Mellacheruvu et al., 2015).

In this work, we found that under the stresses, the contents of MDA and H_2_O_2_ in the transgenic poplars were lower than that in the WT. In addition, evidence from histochemical staining demonstrated that ROS accumulation in the transgenic poplars was lower than that in the WT. These lines of evidence suggested that *ERF38* gene might decrease membrane lipid peroxidation, in order to maintain the stability of membrane structure and reduce ROS accumulation, thereby improving poplar tolerance to salt and osmotic stresses.

In contrast, POD and SOD are the most important antioxidant enzymes to help eliminate extra ROS in plants, so that ROS in plants are maintained at a low level, to increase the tolerance of plants under adverse conditions (Chen et al., 2004; Mittler et al., 2004; Feng et al., 2016). In the present study, compared to the WT poplars, the transgenic poplars possessed higher POD and SOD activities and the expression of POD and SOD-related genes was significantly up-regulated in the transgenic poplars under the two stresses, suggesting that the *ERF38* gene can increase the relevant antioxidant enzymes in the transgenic poplars in response to salt and osmotic tolerance. Moreover, plants can produce osmotic regulators, such as proline and soluble proteins, in response to abiotic stresses (Wani and Gosal, 2011; de Freitas et al., 2019). High levels of proline and soluble protein are beneficial to maintenance of cell structure and function in plants, thus improving plant tolerance (Li et al., 2011; Xiong et al., 2018). In addition, proline in plants can effectively synergize with SOD enzymes to scavenge the ROS (Szabados and Savourcb, 2010; Rejeb et al., 2014). Our data indicated that transgenic poplars produce more proline and soluble protein than WT poplars, which provides another evidences supporting the function of *ERF38* gene in salt tolerance and osmotic tolerance.

ABA is an important signal molecule for abiotic stress, and it has important biological functions in abiotic stresses, such as salt and drought (Wang et al., 2011; Leon et al., 2014; Saxena et al., 2016). Abiotic stress factors can induce the synthesis of ABA (Xiong et al., 2002). It can change the stomata closure to regulate the transpiration rate and regulate the synthesis of osmotic substances to improve the tolerance of plants (Liu et al., 2005; Tuteja, 2007). The expression of some genes can also increase the plant tolerance by participating in the ABA signaling pathway (Li et al., 2019a; Li et al., 2019b). All evidence suggests that ABA is a key hormone in response to abiotic stress. In the present study, we found that the expression levels of ABA-responsive genes in transgenic poplars had a significantly elevated compare to WT poplars under PEG-6000 stress condition. However, we did not investigate whether or not the *ERF38* gene can regulate the ABA signaling pathway, therefore it will be incorporated into future studies, in order to scrutinize molecular functions of the *ERF38* gene.

## Conclusions

In the study, we isolated a salt-inducible ERF gene, *ERF38*, from *P. alba × P. glandulosa*, followed by transferring it into poplar. Evidence from yeast-one-hybrid indicated that ERF38 protein can specifically bind to the DRE element. And the transgenic poplar lines over-expressing *ERF38* gene have advantages in the morphological, physiological, and biochemical traits, compared to WT poplars. Furthermore, expression levels of several POD and SOD-related genes were significantly higher in the transgenic lines than that in the WT. All the results indicated that over-expression of *ERF38* gene can improve salt and osmotic tolerance of transgenic poplar by regulating the expression of stress-related genes.

## Data Availability Statement

All datasets for this study are included in the article/ **Supplementary Material**.

## Author Contributions

TJ and BZ designed research. ZC conducted experiments and data analysis and wrote the manuscript. XZ and KZ performed in data analysis. RL and WY revised the manuscript. All authors read and approved the manuscript.

## Conflict of Interest

The authors declare that the research was conducted in the absence of any commercial or financial relationships that could be construed as a potential conflict of interest.
