# Supplementary material for: Over-Expression of ERF38 Gene Enhances Salt and Osmotic Tolerance in Transgenic Poplar
Source: Front Plant Sci. 2019 Nov 4;10:1375. doi: 10.3389/fpls.2019.01375 (PMC6843637; doi:10.3389/fpls.2019.01375)
Supplement: Supplementary file 1 [file Table_1.docx]

**Supplementary Tables**

**Supplementary Table S1 Primer information**

| Primer pairs | Forward primers | Reverse primers |
| --- | --- | --- |
| ERF38 | GCGTCTAGAGGTGGCGACGACTCCTGGAGCCCGATGGTGAGAGAGAGAAGGGAGAGA | GCGGAGCTCCATTACCAGTTGGTCTGGTGTCAACACGGGACAGTAAAGAAGAAGAAG |
| ERF38-GFP | GGGTCGACTGACTAGTATGGTGAGAGAGAGAAGGGAGA | TGCTCACCATACTAGTAACTGTCCACACACCAGATG |
| D-ERF38 | CGACAGAGATATTGCCTCCC | CCGCGCAGATTAGGGAGGTT |
| Action | ACCCTCCAATCCAGACACTG | TTGCTGACCGTATGAGCAAG |
| pBI121 | CCATCGTTGAAGATGCCTCTGC | CTCTTCGCTATTACGCCAGCTG |
| SZ-ERF38 | GGAATTCATGGTGAGAGAGAGAAGGGAGA | GGGATCCTTAAACTGTCCACACACCAGAT |
| DRE | AGCTTTACCGACATTACCGACATTACCGACATGAGCT | TATGTCGGTAATGTCGGTAATGTCGGTAG |
| DRE-M1 | AGCTTTATTGACATTATTGACATTATTGACATGAGCT | TATGTCGGTAATGTCGGTAATGTCGGTAG |
| DRE-M2 | AGCTTTACCTTCATTACCTTCATTACCTTCATGAGCT | TATGAAGGTAATGAAGGTAATGAAGGTAG |

The primer of ERF38 was used to amplify the coding sequence of *ERF38* gene, and the *ERF38* gene was inserted into the vector PBI-121 by insertion of the *XbaI* and *SacI* restriction sites to form an overexpression vector. ERF38-GFP was used to develop a fusion construct (35S::ERF38::GFP) and the underline indicated the restriction site *SpeI.* D-ERF38 was used to measure the relative expression level of transgenic poplars according qRT-PCR. Action is an internal reference primer. The role of the pBI121 primer is to detect transgenic poplars. SZ-ERF38 was ligated into pGBKT7 and PGADT7 vectors by inserting *EcoRI* and *BamHI* cleavage sites, respectively, for yeast two-hybrid auto activation assay and yeast one-hybrid effect vector construction.

**Supplemental Table S2 Stress-related genes and primer sequences**

| Function | Gene ID | Forward primers (5’-3’) | Reverse primers (5’-3’) |
| --- | --- | --- | --- |
| POD1 | Potri.016G084800 | GTCTTATGCTGACTTCTACC | AATCCAGAACGCTCCTTGTG |
| POD2 | Potri.015G003500 | GGTTCTGTGCTTCTCGACTC | TGTGCCATCTCTTCTTCCAG |
| POD3 | Potri.007G067200 | GCTGAGTCCATTGTTAGATC | GCCTGCCTCTCAATGGAACG |
| POD4 | Potri.016G132700 | CCTCATCAACCATCACTACC | TAGTATTTGCTGGTGCTGTC |
| POD5 | Potri.007G053400 | GTTCCATCTCTTACTTGTTC | CTTGTTGGTTGCAGTGGAGG |
| SOD1 | Potri.005G089600 | GAGTAAGAGGACAGTAGAGG | ATGTCTCCTCCTCCTGGTTG |
| SOD2 | Potri.009G005100 | CTAATGTTGAAGGCGTCGTC | ACGCATCCATTTGTTGTGTC |
| SOD3 | Potri.013G092600 | GAATCTCACTCCTGTCCAAG | CAACACCAAGTAATGGAAC |
| SOD4 | Potri.006G049100 | CTCTCATTCTCCTCTCCGTG | CCAGGCACACCACCAACTTG |
| SOD5 | Potri.009G005100 | CGCTGCCAAGAAGCAACCAC | ACGACGCCTTCAACATTAGA |
| *RD29B*  *ZmRD22B*  *ZmPTF1*  *PtPYRL1* | Potri.012G141300  Potri.018G036700  Potri.006G135600  Potri.001G142500 | CAGTAGGATTGGAGGAGGAT  GAACTGCTGTAAATGTAGGG  GCAATGGAAGGGAACAATCT  CTGTTGGCATTAGATTTGTG | GTTATTCCTGCTTCTTTCCC  CCTTTCCCAGAGTTTACATT  GAAGAAACACCCTCATAAGA  GATAAGAGTGGGGTTTTTGT |

**Supplementary Figure**

**Supplementary Figure S1**

**
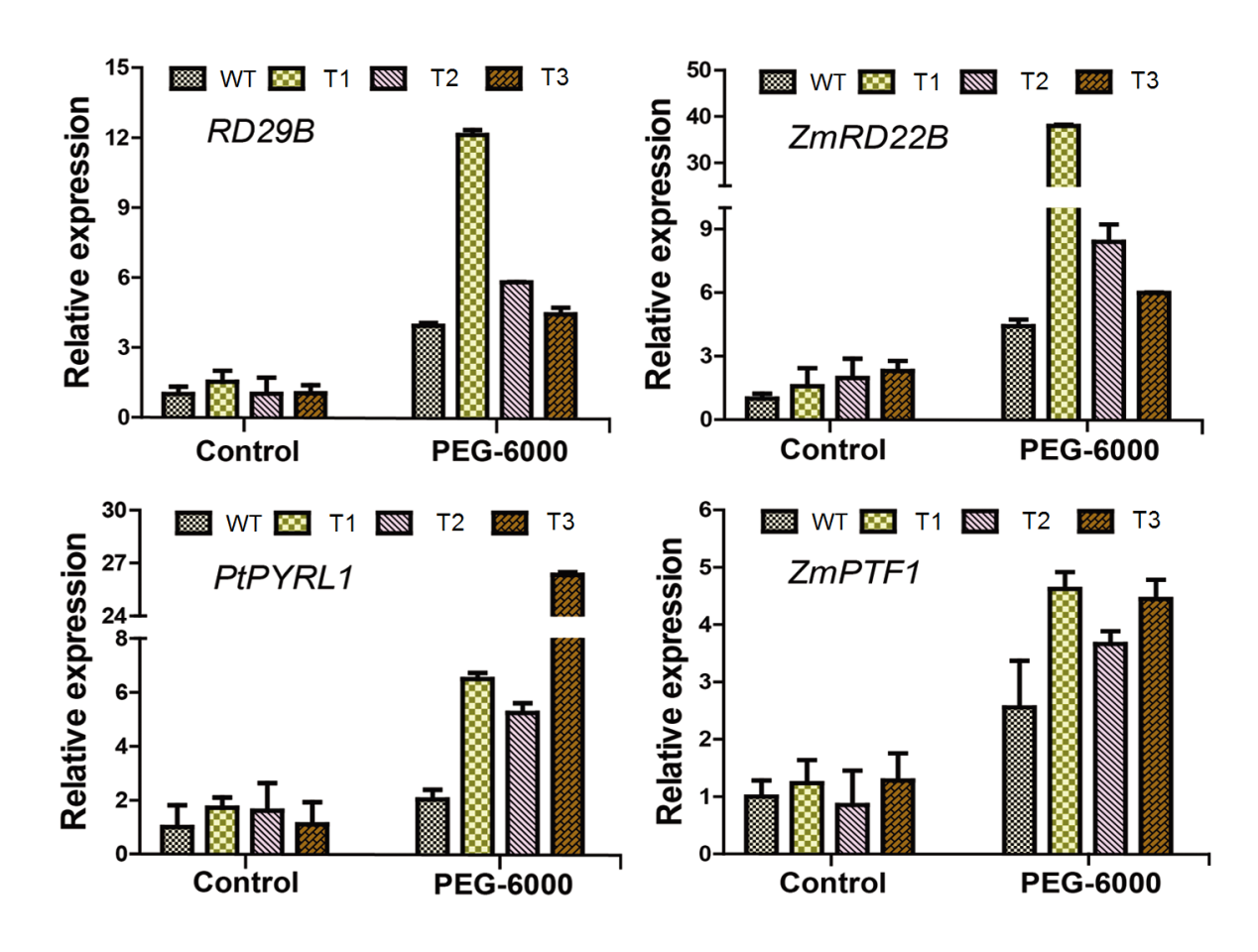
**

**FIGURE S1|** Analysis of genes expression under polyethylene glycol stresses. T1-T3: different transgenic poplar lines; WT: wild type poplar; the control is water.
